# IgA nephropathy in a laboratory worker that progressed to end-stage renal disease: a case report

**DOI:** 10.1186/s40557-016-0118-z

**Published:** 2016-08-08

**Authors:** Bokki Min, Gyuree Kim, Taesun Kang, Chungsik Yoon, Sung-il Cho, Domyung Paek

**Affiliations:** 1 Program of Occupational and Environmental Medicine, Graduate School of Public Health, Seoul National University, Gwankakro 1, Gwanak-gu, Seoul 151-600 Republic of Korea; 2 Department of Environmental and Safety Engineering, Ajou University, 206 Worldcup-ro, Yeongtong-Gu, Suwon, 443-749, Republic of Korea; 3 Department of Environmental Health, Graduate School of Public Health, Seoul National University, Gwankakro 1, Gwanak-gu, Seoul 151-600 Republic of Korea

**Keywords:** IgA nephropathy, End-stage renal disease, Organic solvent, Occupational exposure, Laboratory worker, Toluene, Dichloromethane

## Abstract

**Background:**

IgA nephropathy (IgAN) is the most common form of glomerulonephritis, a principal cause of end-stage renal disease (ESRD) worldwide. The mechanisms of onset and progression of IgAN have not been fully revealed, and epidemiologic studies have yielded diverging opinions as to the role of occupational exposure to organic solvents in the initiation or worsening of IgAN. As the authors encountered a laboratory worker with IgAN that progressed to ESRD, we present a case report of IgAN progression due to dichloromethane exposure along with a review of literature.

**Case presentation:**

A 41-year-old male laboratory worker began to experience gross painless hematuria after two years of occupational exposure to toluene. Although clinical follow-up was initiated under the impression of IgAN based on clinical findings, the patient continued to work for four more years in the same laboratory, during which he was in charge of laboratory analysis with direct exposure to a high concentration of dichloromethane without proper protective equipment. During that time, his renal function rapidly worsened and finally progressed to ESRD 10 years after the first clinical symptoms. The result of exposure assessment through reenactment of his work exceeded the occupational exposure limit for dichloromethane to a considerable degree.

**Conclusions:**

The causal association between occupational solvent exposure and IgAN is still unclear; therefore, this case report could be used as a basis to support the relevance of occupational solvent exposure to IgAN and/or its progression. Early intervention as well as close monitoring of laboratory workers exposed to various organic solvents is important to prevent or delay the progression of glomerulonephritis to ESRD in the occupational setting.

## Background

IgA nephropathy (IgAN) is a glomerular disease characterized by IgA deposition in the glomerular mesangium of the kidney, which was first reported by Berger et al. in 1968 [1]. IgAN is the most common form of glomerulonephritis in the world, accounting for about 30–45 % of all primary glomerular diseases [2]. IgAN can occur in all age groups, but is mainly found in patients in their 10s–30s, and it is more common in males, with a male to female gender ratio of 2:1 [3, 4]. The disease can be diagnosed through biopsy by visualization of IgA deposition in the glomerular mesangial area using immunofluorescence microscopy [2, 4]. IgAN presents with macroscopic hematuria in about 40 to 45 % of patients, with microscopic hematuria and proteinuria in about 35 to 40 %, and with nephrotic syndrome or acute renal failure in the remainder [3, 5]. While IgAN has an indolent course, about 30 % of patients will reach end-stage renal disease (ESRD) after 20 years, particularly in those who present with hypertension, heavy proteinuria or renal insufficiency [3–5]. Still no treatment is known to modify mesangial deposition of IgA, and available treatment options are directed to mostly at downstream immune and inflammatory events that may lead on to renal scarring [4]. Although the etiology and pathophysiology of IgAN have not yet been clearly identified, accumulating evidence suggests that the onset and progression are caused by interactions between various genetic and environmental factors [6–9]. Among them, occupational risk factors such as exposure to organic solvents have been suggested as probable causes that may induce [7, 8, 10] or aggravate IgAN [11–13].

The present case report is the first report in Korea suggesting the possibility that occupational exposure to organic solvents such as toluene and dichloromethane, commonly used in laboratories and industrial fields, can act as a risk factor for the onset and/or progression of IgAN. The present case report attempted to investigate the association of occupational solvent exposure and IgAN based on patient clinical information, inspection of the working environment, exposure assessment, and review of related literature, by which the authors aimed to contribute to the approach and prevention of other cases that may occur in the future.

## Case presentation

### Medical assessment

#### Patient

Male, 55 years old (at the time of clinic visit for the assessment of occupational disease).

#### Chief complaint

End-stage renal disease with histories of gross painless hematuria and chronic fatigue.

#### Present illness

He first experienced the symptom of gross painless hematuria in June 2000, when he was 41 years old. Urinalysis performed in A hospital in December 2000 identified hematuria and proteinuria, while the serum creatinine (Cr) concentration was in the normal range (red blood cells [RBC]: Many, Cr: 1.3 mg/dL, 24 h urine protein: 266.2 mg/day). The patient was hired in the current laboratory institute in 1987, when he was 28 years old. Before the first episode of gross hematuria, for 2 years and 7 months (February 1998 to August 2000), corresponding to ages 39 to 41 years for the patient, his work involved analysis of the dioxin contents in exhaust gas at an incineration facility by a pretreatment method using toluene in the laboratory.

In July 2001, the patient was clinically diagnosed with IgAN without a biopsy in B hospital, and clinical follow-up was initiated. Although the patient was in follow-up after the clinical diagnosis with IgAN, he performed work involving analysis of total petroleum hydrocarbon (TPH) in soil samples using a dichloromethane as a solvent again in the laboratory for 4 years and 4 months (March 2005 to June 2009), corresponding to ages 46 to 50 years. During the TPH analysis work, the patient was continuously exposed to dichloromethane in a laboratory under conditions of poor ventilation without appropriate protective devices such as a respirator and gloves. The Cr level in the patient’s blood test started to increase from April 2006 (April 2006, Cr: 1.7 mg/dL; November 2007, Cr: 2.2 mg/dL), followed by exacerbation of the systemic fatigue symptom, so he was admitted to C hospital in March 2008, when he was 49 years old, and diagnosed with IgAN by a biopsy. Since 2008, the patient’s renal function rapidly deteriorated (June 2009, Cr 3.9 mg/dL; September 2009, Cr 5.9 mg/dL; April 2010, Cr 8.4 mg/dL) and finally progressed to ESRD, and peritoneal dialysis was begun in June 2010 when he was 51 years old. As of December 2014, he was in follow-up after kidney transplantation at the age of 55 years, after 13 years had passed from the initial clinical diagnosis with IgAN.

#### Clinical therapeutic course

The patient began to take an angiotensin II receptor blocker (ARB) after clinical diagnosis in 2001, and thereafter, the patient’s blood pressure, serum BUN/Cr level, and urine protein/creatinine ratio (PCR) have been measured at regular outpatient follow-ups. He incorporated life style modifications for weight management and abstained from alcohol, and started a low-salt, low-protein diet with the initiation of medication. When his serum Cr increased to 1.7 mg/dL in April 2006, he began taking an angiotensin-converting enzyme inhibitor (ACEi), HMG-CoA reductase inhibitor (statin), omega-3, and blood pressure medication, and his blood pressure, renal function, and proteinuria level were closely monitored. However, the patient’s renal function and proteinuria level began to deteriorate in 2008, and he ceased analytic work in the lab and changed to a desk job in June 2009. Despite starting oral steroid therapy in September 2009, no particular benefit was observed, and the patient began peritoneal dialysis in June 2010, eventually undergoing renal transplantation in December 2014.

#### Past history

The patient had a history of diagnosis at the age of 21 with pleural effusion and tuberculous pleurisy, which was completely cured after a two-year course of medication. Other than that, his past medical history is essentially unremarkable. There is no evidence of preexisting hypertension, cardiovascular disease, diabetes mellitus, and there is no preexisting history of drug abuse, alcoholism or medication abuse. The patient received regular occupational health examinations two times (in 1997 and 1999) before the onset of his first symptom of hematuria and had no abnormal findings on the corresponding health examinations, in particular, on kidney-related examinations such as hematuria and proteinuria.

#### Family history

His family history is essentially unremarkable. When the family history of the patient was thoroughly examined, no first-degree relatives had any history of specific disease including kidney disease.

#### Social history

The patient was not smoking and his alcohol intake history was 3–4 times a month, with about 0.5 bottle of soju each time.

#### Pathologic examination

Eight years after the first clinical symptom appeared, the patient was finally confirmed to have IgAN (subclass V, by Haas histological classification [14]) through a kidney biopsy, in March 2008 when he was 49 years of age. The biopsy findings were as follows: “Tubules reveal focal moderate atrophy or loss with focal moderate infiltration of mononuclear cells and fibrosis in interstitium. Fibrointimal thickening of small arteries is noted.”

#### Lab test follow-up

**Fig. 1:**
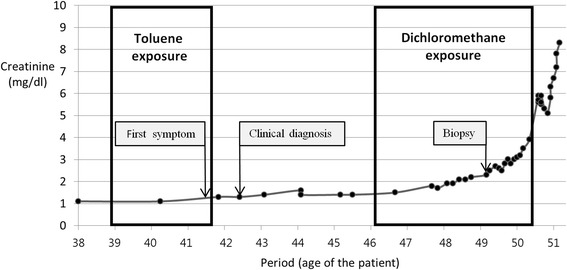
Changes in creatinine (Cr) levels according to period of exposure to organic solvents and lapse of time; Dots on the line indicate time points when Cr levels were measured. The first and second measurements of Cr levels were made in February 1997 and May 1999, when the patient was 38 and 40 years old, respectively. Cr levels were measured every 6–12 months after 1999, every 2 months starting in 2007 (when the patient was 48 years old), and every month starting in 2008 (when the patient was 49 years old)

The authors reviewed all the medical records of the patient, focusing on the assessment of all serum Cr test results on every medical record from February 1997, when the patient first underwent health examination since the beginning of the job. As a result, we found that his serum Cr level rapidly increased from the period when he was exposed to dichloromethane for 4 years and 4 months (March 2005 to June 2009) (Fig. 1).

### Occupational assessment

#### Occupational history

The first job of the patient was environmental design-related office work in H company, where he worked for 3 years from 1984, when he was 25 years of age, and he transferred to the current job in May 1987, when he was 28 years of age. Except for the period when the patient performed general office work, he was continuously involved in sample analysis work in a laboratory, in which periods and tasks in which he was exposed to organic solvents are as follows: 1) Dioxin analysis using toluene pretreatment in the laboratory for 2 years and 7 months (February 1998 to August 2000), since 39 years of age; and 2) TPH analysis of soil by pretreatment using dichloromethane for 4 years and 4 months (March 2005 to June 2009), since the age 46 (Table 1).

**Table 1:** The patient’s history of occupational exposure to organic solvents with disease progression

Duration	Task	Possible exposure	Disease history
Feb 1998 to Aug 2000	Dioxin analysis	Toluene	Jun 2000: Hematuria onset Jul 2001: Clinical diagnosis of IgAN Jul 2001: Clinical follow-up starts
Mar 2005 to Jun 2009	TPH analysis	Dichloromethane	Oct 2006: Renal function exacerbation Mar 2008: Renal biopsy confirmed Jun 2010: Peritoneal dialysis starts Dec 2014: Kidney transplantation

Among these tasks, according to our preliminary study, TPH analysis work using dichloromethane was suspected as the work in which the patient was exposed to organic solvents in a high concentration for a long period of time without proper protective devices; considering whether the presence of relevant data and the possibility of reconstruction of working environment, the present case report was focused on the TPH analysis work in detail for evaluation of occupational exposure, during which the patient’s IgAN rapidly progressed to ESRD.

#### Job analysis

**Fig. 2:**
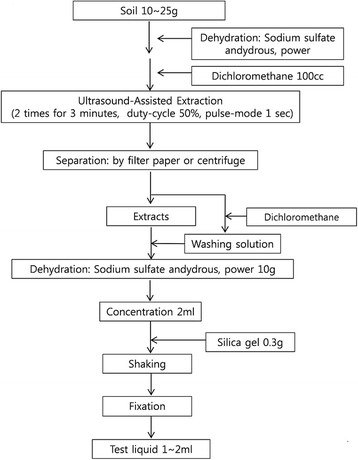
The Standard Test Method of Soil Contamination on TPH pretreatment by the Ministry of Environment of Korea (Ultrasound-Assisted Extraction method)

The pretreatment work in TPH analysis of soil that the patient performed was conducted according to the ultrasound-assisted extraction method of TPH in item number 18 of the Standard Test Method of Soil Contamination by the Ministry of Environment of Korea, and its schematic process is presented in Fig. 2. The patient reported 4–8 h of daily exposure and described symptoms of dizziness, headaches, and nausea during the pretreatment process. Despite consideration of the general office tasks he did in parallel with TPH analysis work, when the working hours of TPH analysis were estimated based on the descriptions of the patient and coworkers during the 4 years and 4 months, the exposure frequency to dichloromethane of the patient was at least 4 h/day, so the exposure frequency was speculated to be quite high.

#### Exposure environments

The authors investigated the work environment of the laboratory institute and reviewed all past work environment data measurements. Pretreatment process in TPH analysis was performed in ‘Room A’ before 2008, where the door always remained closed during experiments due to the noise generated in the pretreatment process. According to the descriptions by the patient and coworkers, a local ventilation system had been installed for ventilation, but ventilation conditions were poor and workers often performed pretreatment works without the provision of protective devices such as a respirator and gloves. The location for pretreatment work of TPH analysis was changed in 2008 to ‘Room B’ (123.8 m^2^) with an area about 4 times larger than ‘Room A’ (30.1 m^2^). In addition to a preexisting ventilation system in ‘Room B’, an additional local ventilation system was installed in 2010, and three ultrasound-assisted extractors (the major source of dichloromethane) were placed in the newly installed hood facilities.

According to the results of working environment measurement in 2011, however, levels of dichloromethane exposure in two laboratory workers who performed the corresponding pretreatment work using dichloromethane in ‘Room B’ were 90.239 ppm and 53.045 ppm, exceeding 50 ppm, the time-weighted average (TWA) suggested in Korean Industrial Safety and Health Act (Table 2).

**Table 2:** Exposure assessment history of the corresponding workplace for pretreatment work

Year	Measurement result (ppm)	
	Dichloromethane	Mixed organic compounds
~2009	No data	No data
2010	11.19	0.22
2011	90.24	1.80
2011	53.05	1.06
OEL^a^ (TWA^b^)	50	1

^a^OEL: Occupational Exposure Limit

^b^TWA: Time-Weighted Average

#### Exposure assessment through reenactment

**Fig. 3:**
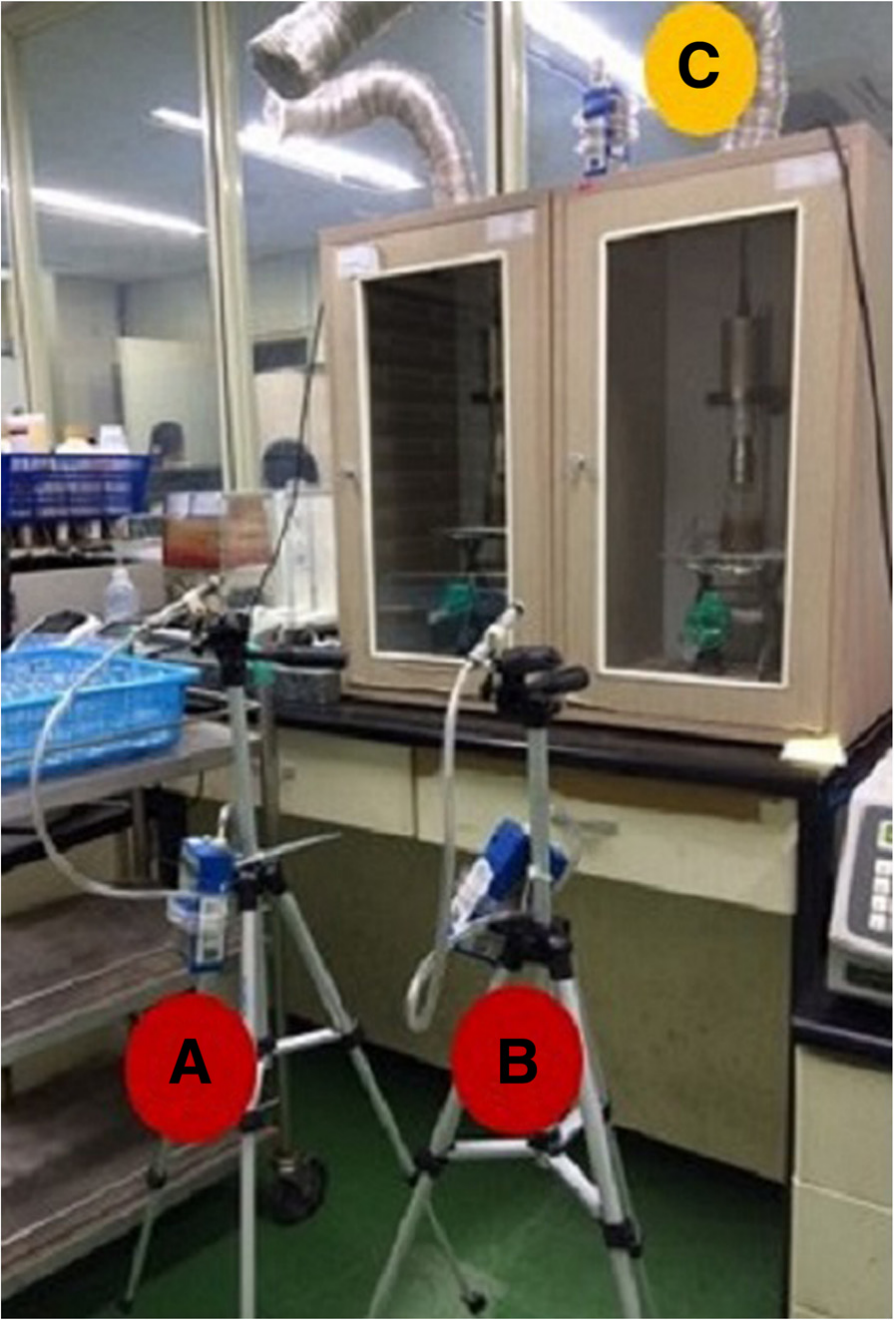
Site of exposure reenactment (Two ultrasound-assisted extractors are located inside separate hood chamber). Measurement position **a**. In front of Extractor 1. Measurement position **b**. In front of Extractor 2. Measurement position **c**. Above the two Extractors

The work space of ‘Room A’, where the work was actually carried out at that time, was accurately restored and the process of ultrasound-assisted extraction using dichloromethane was reenacted repeatedly for about 2 h following the same method that the patient had actually performed (Fig. 3). Predictable errors were minimized by referring to coworkers who performed the work with patient and managers. For measurement, samples in the air during the work of ultrasound-assisted extraction were collected. Detailed measurement and analysis of dichloromethane in the air was performed based on the 1005 method of the US National Institute of Occupational Safety & Health (NIOSH). As a result of the measurement, the time-weighted average concentrations of dichloromethane at the corresponding spots where pretreatment workers were positioned were 254 and 181 ppm, respectively, and the ranges of short-term exposure concentration were 68–374 ppm and 88–359 ppm, respectively (Table 3).

**Table 3:** Results of exposure reenactment of dichloromethane

Measurement point	Duration (min)	Short-term exposure concentration (ppm)	Time-weighted average concentration (ppm)
A. Ultrasound-assisted extractor 1 (Respiratory location)	23	68	254
	26	200	
	21	359	
	26	301	
	18	374	
B. Ultrasound-assisted extractor 2 (Respiratory location)	23	91	181
	26	157	
	21	88	
	26	228	
	18	359	
C. Above the ultrasound-assisted extractors	113	-	179

OEL (Occupational exposure limit): Time-weighted average 50 ppm (the Korean Industrial Safety and Health Act), short-term exposure limit 125 ppm (OSHA)

These results suggest that the actual respiratory exposure concentrations of the patient to dichloromethane in ‘Room A’ during the pretreatment work for TPH analysis during the period of March 2005 to June 2009 should have been higher than the time-weighted average (50 ppm) set by the Korean Industrial Safety and Health Act and the short-term exposure limit (125 ppm) set by OSHA (Occupational Safety and Health Administration). Therefore, it can be surmised that the patient had been regularly exposed to a high concentration of dichloromethane in ‘Room A’ for 52 months.

## Discussion

A number of studies have reported kidney toxicity of organic solvents and an association with glomerulonephritis. Miller et al. [15] reported a case of acute tubular necrosis accompanied by acute renal failure, myoglobinuria, and liver enzyme elevations that were caused by inhalation exposure to dichloromethane. They conclude that dichloromethane may have potential as both a hepatotoxic and nephrotoxic agent when inhaled at high concentrations over an extended period of time [15]. Ana-Lilia et al. [16] reported that shoe-workers who were occupationally exposed to toluene-based glues had a higher risk of renal glomerular and/or tubular damage than the control group. Ravnskov et al. [17] conducted a meta-analysis of 14 case–control studies and reported that the combined data support the hypothesis that hydrocarbon exposure increases the risk of chronic renal disease, although no specific hydrocarbons were identified in these reviews. In addition, there have also been other case reports [18], epidemiological studies [8, 19, 20] and animal-experimental studies [19, 21] reporting associations between solvent exposure and onset of glomerulonephritis or deterioration of renal function. On the other hand, a few studies [22, 23] reported negative results on the association between solvent exposure and the risk of chronic glomerulonephritis.

In the clinical literature that specifically reported IgAN in relation to occupational exposure, several case studies reported the onset of IgAN due to exposure to organic solvents [24, 25], in addition to cadmium [24, 26, 27] and silica [28] (Table 4). Albrecht et al. [25] reported the case of a 28-year-old male pipe plumbing worker who developed IgAN after occupational exposure to organic solvents. The corresponding patient was exposed to organic solvents during 9 years and 6 months of pipe plumbing work, resulting in the symptom of gross hematuria, and he was diagnosed with IgAN by a biopsy [25]. Working environment measurement identified that the patient mainly carried out pipe plumbing tasks in closed bathrooms, during which he was exposed to a high concentrations (389–757 ppm) of tetrahydrofuran (THF), a component of polyvinyl chloride (PVC) pipe cement (the short-term exposure limit for THF set by the NIOSH is 250 ppm) [25]. The authors stated that a predisposition toward IgAN in this patient could be exacerbated by massive short-term exposure to the solvent [25].

**Table 4:** Case reports of IgAN associated with occupational solvents exposure

Author(s) (year)	Gender/Age at diagnosis	Occupation	Type of solvent	Exposure duration	Biopsy confirm	Prognosis
Albrecht et al. [25] (1987)	Male/28y	A plumber, working with solvent-based pipe cement	tetrahydrofuran (THF)	9 years and 6 months	+	not identified
Fernandez et al. [24] (2010)	Male/47y	A pesticide manufacturer	various organic solvents (toluene, xylene, acetone, cyclohexanone, etc.)	23 years	+	progression to CRF in 2 years after diagnosis

Meanwhile, Fernandez et al. [24] introduced a case of IgAN in a 50-year-old male who was exposed to various types of organic solvents for 23 years. The male was continuously exposed to organic solvents occupationally for an additional two years even after diagnosis with IgAN, resulting in progression to CRF stage 3 in two years after diagnosis with IgAN [24].

Literature searches on epidemiologic studies that analyzed correlations between IgAN and occupational solvent exposure resulted in a total of three case–control studies reported to date [10, 11, 29] (Table 5). First, Porro et al. [10] investigated occupational and non-occupational exposures to organic solvents in 60 chronic glomerulonephritis patients diagnosed by biopsy in the Bari area of Southern Italy and a control group (60 nephrolithiasis and 60 traumatic fracture patients) from 1983 to 1987. The odds ratio of chronic glomerulonephritis for patients occupationally exposed to solvents was 3.9 (95 % confidence interval [CI] 1.64–8.33), and a logistic regression model showed a dose–response relationship of occupational exposures to solvents and glomerulonephritis [10]. When IgAN patients (*n* = 27) were separately evaluated, an increased risk was found for both total (Relative risk [RR] = 3.5, 95 % CI 1.18–12.18) and occupational exposure (RR = 4.25, 95 % CI 1.18–16.36) [10].

**Table 5:** Epidemiological studies investigating the association of occupational solvent exposure and IgAN

Author(s) (year)	Study design	Subjects	Outcome	Period	Population Total GN^a^/IgAN	Results^e^
Porro et al. [10] (1992)	case–control study	60 biopsy-proven chronic GN^a^ patients in a University hospital in Italy (including 27 IgAN patients)	onset of IgAN	1987–1990	60/27 (reference:120)	·An increased risk of IgAN was found for occupational solvent exposure group. ·RR^b^ of IgAN for occupational solvent exposure ; 4.25 (1.18–16.36)
Stengel et al. [11] (1995)	case–control study	298 biopsy-proven GN^a^ patients in 5 hospitals in Paris (including 116 IgAN patients)	aggravation of IgAN to CRF	1989–1991	298/116 (reference:298)	·Among males, clear association was observed between CRF and high exposure to solvents for IgAN; OR^c^ = 3.5 (1.0–11.8), *P* < 0.05. ·The OR increased with duration of exposure; OR = 5.6 (1.3–24.1) for ≥10 years exposure, (*P* = 0.02). ·No relationship was observed for cases without CRF.
Wakai et al. [29] (1999)	case–control study	94 biopsy-proven IgAN patients in medical centers in Japan	onset of IgAN	1997–1999	94/94 (reference:185)	· Work-related exposure to organic solvents was found not to be associated with the risk for IgAN; OR = 0.55 (0.27–1.12), (*P* < 0.10)
Jacob et al. [12] (2007)	retrospective cohort study	338 non-ESRD patients in Paris (including 194 IgAN patients with biopsy confirm)	aggravation of IgAN to ESRD	2002–2004	338/194	·Solvent exposure was associated with faster progression of IgAN to ESRD, HR^d^ for IgAN is 2.6 (1.3–5.5) for high exposure versus none (*p* < 0.05). ·There was a trend increasing HR with exposure duration before and its persistence after diagnosis.
Jacob et al. [13] (2007)	retrospective cohort study	269 patients with non-ESRD and biopsy-proven primary GN^a^ diagnosis between 1994 and 2001 in Paris and suburbs (including 167 IgAN patients)	aggravation of IgAN to ESRD	2002–2004	269/167	·This study showed the potential role of toluene and xylene, some petroleum products, ketones and possibly dichloromethane in the progression of GN^a^ to ESRD.

^a^GN, Glomerulonephritis; ^b^RR, Relative Risk; ^c^OR, Odds Ratio; ^d^HR, Hazard Ratio; ^e^(··), 95 % Confidence Interval

In contrast, a case–control study that was conducted by Wakai et al. [29] with Japanese subjects to investigate a correlation between diagnosis of IgAN and occupational exposure to organic solvents showed an odds ratio of 0.55 (95 % CI 0.27–1.12), which was not statistically significant [29]. The authors commented that they might have failed to find a positive association because of the few number of patients with advanced renal failure, and also pointed out that another possible limitation was the methodological difficulty of measuring exposure to organic solvent [29].

Meanwhile, Stengel et al. [11] performed a case–control study to investigate a correlation between organic solvent exposure and CRF with 116 IgAN patients in five hospitals in Paris. As a result, among males, a clear association was observed between CRF and high exposure to organic solvents for IgAN patients (OR = 3.5, 95 % CI 1.0–11.8) [11]. In addition, the odds ratio increased with duration of exposure [11]. On the other hand, patients with IgAN who had normal renal function showed no correlation with organic solvents, so these results were interpreted to suggest that organic solvents contributed to deterioration of renal function to some degree rather than to the diagnosis itself of IgAN [11].

Meanwhile, in 2007, Jacob et al. [12] conducted the GN-PROGRESS cohort study; the authors investigated occupational risk factors for the progression of glomerulonephritis to ESRD. Using a cohort study design, they showed that solvent exposure was indeed associated with faster progression to ESRD with IgAN (Hazard ratio [HR] = 2.6, 95 % CI 1.3–5.5) [12]. In patients with IgAN, there was a trend in increased hazard ratios with exposure duration before and its persistence after diagnosis [12].

In a subsequent study, using data from the GN-PROGRESS cohort, Jacob et al. [13] therefore systematically investigated the risk of progression to ESRD, by solvent-exposed job category, type of solvent-containing products, and by solvent or solvent family. In this analysis [13], they focused on the 269 patients with either IgAN or membranous nephropathy. Among solvents, the highest risks were found for: toluene/xylene (HR = 5.1, 95 CI 1.8–14.8), gasoline, fuel and gas-oil (HR = 8.6, 95 CI 2.7–27.4), and ketones (HR = 13.3, 95 % CI 1.4–123.5). Most interestingly, they also observed an excess risk for any exposure level to methylene chloride, also known as dichloromethane (HR = 6.4, 95 % CI 1.7–24.8) [13]. In another occupational cohort study in US aircraft workers, Radican et al. [30] pointed to the risk of all-cause ESRD associated with trichloroethylene, 1,1,1-trichlorethane, dichloromethane, and JP4 gasoline.

In summary, regarding the relationship between occupational solvent exposure and IgAN in the literature, all reviewed studies [11, 12] consistently reported significant correlations at least between solvent exposure and the progression of preexisting IgAN to CRF or to ESRD, though study results regarding the onset of IgAN [10, 11, 29] were inconsistent (Table 5). In particular, recent cohort study results [12, 13, 30], showing the potential role of toluene and dichloromethane in the progression of IgAN to ESRD supports our hypothesis that the IgAN of this case report may have progressed more rapidly to ESRD due to occupational solvent exposure.

Although it can occur at any age, IgAN mainly occurs in young adults (16–35 years old) [3, 31–33]. In a Chinese cohort study of 1,115 patients with IgAN [34], the mean age at time of initial clinical manifestations and renal biopsy were 31 ± 9, and 33 ± 9 years old, respectively. For the patient in the present case, gross hematuria was first experienced at 41 years of age in June 2000, and the serum Cr level at that time was 1.3 mg/dL, corresponding to a normal level. In July 2001, he was given a clinical diagnosis of IgAN (not biopsy confirmed), which was when the patient was 42 years old. This is much older than the usual age for initial clinical presentation and diagnosis of IgAN compared to the mean age of diagnosis (10–30 years) reported in other studies [31, 32, 34], so it was suspected that this case could be different from the general cases that were diagnosed at earlier ages, at about 10–30 years, mostly due to internal or genetic factors.

For the natural history of IgAN, the actuarial renal survival rate for 10 years after diagnosis or after a biopsy is a significant parameter for prognosis prediction of IgAN [31, 35]. According to a meta-analysis by Coppo [35], the actuarial renal survival rate for 10 years after diagnosis with IgAN or after a biopsy was mostly about 80–90 % in Europe, Asia and Australia. Another study conducted in United Kingdom, using Medical Research Council’s Glomerulonephritis Registry [36], reported that the 10-year cumulative renal survival rate was 83.3 %. Le et al. [34] reported a cohort study with 1,115 IgAN patients in China, in which renal survival rates after diagnosis with IgAN were 83 at 10 years, 74 at 20 years, and 64 % at 30 years. In contrast to the actuarial renal survival rate of 80–90 % for 10 years reported in the literature [31, 34–36], this patient experienced rapid deterioration of renal function within only 9 years from the initial clinical diagnosis to dialysis. Thus, the prognosis is extraordinarily poor.

On the other hand, studies on factors enabling prediction of the prognosis of IgAN commonly suggested “unfavorable histopathologic findings” such as glomerular sclerosis and interstitial fibrosis, and “severe proteinuria at presentation,” “arterial hypertension at presentation and during follow-up,” and “elevated serum creatinine at presentation” as strong clinical predictors [31, 37–39]. The patient in this case did not have confirmation by biopsy until 8 years after the first onset of symptoms. Since biopsy was performed when histological damages had already progressed significantly, it is difficult to predict the prognosis based on the time points of first onset and first diagnosis based on biopsy findings. Meanwhile, this patient had a serum Cr level of 1.3 mg/dL at the time of initial clinical diagnosis, corresponding to within the normal range, and 266 mg/day of 24 h urine protein, showing that he was not in a severe condition. Furthermore, the patient had neither underlying diseases such as hypertension and diabetes from the past, nor hypertension at the time of diagnosis. In other words, although the patient was in a condition without known strong clinical predictors, the deterioration of his renal function rapidly progressed, unlike the natural progression reported in other studies [31, 34, 36]. In addition, this decline of renal function overlapped with the patient’s period of exposure to dichloromethane between March 2005 and June 2009 (Fig. 1).

In summary, based on a literature review of the natural history of IgAN, the present case has unusual clinical characteristics such as the age of disease onset, speed of progression, and the prognostic pattern. The authors surmised that the unusual characteristics of this IgAN case provide supporting evidence for the relevance of occupational solvent exposure with IgAN and/or its progression.

This case study has several limitations. First, exposure assessment for toluene could not be conducted because there were almost no relevant data remains. The first episode of hematuria, the initial clinical manifestation of IgAN in this patient, actually began after he had been exposed to toluene while performing analysis of dioxin contents in exhaust gas from an incineration facility during a period of 2 years and 7 months (Table 1). According to reports by the patient and co-workers, however, there was no proper ventilator system in the pretreatment room at that time, and workers usually did not wear protective equipment. Thus, it is presumed that there was direct exposure to a high concentration of toluene. Therefore, the authors cautiously suggest that possibilities of a causal relationship between toluene exposure and the onset and/or aggravation of IgAN could not be ruled out in present case. Secondly, despite the attempt to minimize errors by restoration of the past actual working environment at the site of investigation for exposure, there may still be errors in perfect reflection of the past working environment for the patient including ventilation conditions and the circulation of worker’s traffic. Thirdly, the health effects of other chemical substances used in the laboratory where the patient worked, or potential secondary health effects generated while various chemicals were used in combination with organic solvents, were not evaluated. In addition, another drawback in this case report is that biological monitoring markers for dichloromethane such as blood levels of carboxyhemoglobin (COHb) or urinary dichloromethane [40, 41] were not evaluated in the exposure assessment.

## Conclusion

This case report suggests that many laboratory workers may be exposed to high levels of various hazardous chemicals including organic solvents. Although the results are controversial, several previous studies have suggested an association between occupational solvent exposure and the onset of IgAN and/or its progression to ESRD; thus, the IgAN and following ESRD in this patient might have been influenced by prolonged, high exposure to organic solvents. Because IgAN is a major cause of ESRD and there is no disease-targeted treatment, close monitoring to encourage screening for proteinuria, hematuria, or hypertension in workers exposed to organic solvents is crucial for early detection of those with a risk for IgAN. Furthermore, early intervention and elimination of exposure in those diagnosed with glomerulonephritis at an early stage may prevent or delay the progression to ESRD in these workers.

## Abbreviations

BUN, blood urea nitrogen; CI, confidence interval; Cr, creatinine; CRF, chronic renal failure; ESRD, end-stage renal disease; GN, glomerulonephritis; HMG-CoA, 3-hydroxy-3-methyl-glutaryl-Coenzyme A; HR, hazard ratio; IgAN, IgA nephropathy; NIOSH, National Institute of Occupational Safety & Health; OEL, occupational exposure limit; OR, odds ratio; OSHA, Occupational Safety and Health Administration; PVC, polyvinyl chloride; RBC, red blood cells; RR, relative risk; STEL, short-term exposure limit;THF, tetrahydrofuran; TPH, total petroleum hydrocarbon; TWA, time-weighted average
